# Effect of human myoblasts on tenogenic progression in 2D and 3D culture models

**DOI:** 10.1111/joa.14224

**Published:** 2025-01-23

**Authors:** Yoshifumi Tsuchiya, Ching‐Yan Chloé Yeung, Rene B. Svensson, Michael Kjaer

**Affiliations:** ^1^ Institute of Sports Medicine Copenhagen, Department of Orthopedic Surgery, Copenhagen University Hospital – Bispebjerg‐Frederiksberg Copenhagen Denmark; ^2^ Center for Healthy Aging, Department of Clinical Medicine University of Copenhagen Copenhagen Denmark; ^3^ Faculty of Health and Sports Science Doshisha University Kyoto Japan; ^4^ Health and Medical Research Institute, Department of Life Science and Biotechnology National Institute of Advanced Industrial Science and Technology (AIST) Takamatsu Japan

**Keywords:** cell communication, myoblasts, satellite cells, skeletal muscle, tendon, tendon fibroblasts, tendon regeneration, tenocytes

## Abstract

Tendon injuries and disorders associated with mechanical tendon overuse are common musculoskeletal problems. Even though tendons play a central role in human movement, the intrinsic healing process of tendon is very slow. So far, it is known that tendon cell activity is supported by several interstitial cells within the tendon. However, the interplay between the tendon and the adjacent muscle for tendon regeneration and development processes has not been fully investigated. Here, we tested whether factors released from muscle derived myogenic cells (myoblasts) enhance tenogenic progressions of human tendon derived cells (tendon fibroblasts) using two‐dimensional (2D) culture model and a three‐dimensional (3D)‐engineered tendon construct culture model, which mimics tendon regeneration and development. The conditioned media from myoblasts and unconditioned media as control were applied to tendon fibroblasts. In 2D, immunofluorescence analysis revealed increased collagen type I expressing area and increased migration potential when conditioned media from myoblasts were applied. In the 3D‐engineered human tendon construct model, wet weight, diameter, and cross‐sectional area of the tendon constructs were increased in response to the application of conditioned media from myoblasts, whereas the collagen density was lower and mechanical function was reduced both at the functional level (maximum stiffness) and the material level (maximum stress and modulus). These results indicate that myoblast‐derived factors extend collagen expressing area and enhance migration of tendon fibroblasts, while factors involved in the robustness of extra‐cellular matrix deposition of tissue‐engineered tendon constructs are lacking. Our findings suggest that adjacent muscle affects the signaling interplay in tendons.

## Graphical abstract

We investigated whether factors released from muscle derived myogenic cells enhance tenogenic progressions of human tendon derived cells using two‐dimensional (2D) culture model and a three‐dimensional (3D)‐engineered tendon construct culture model. In the 3D‐engineered human tendon construct model, volumes of the tendon constructs increased in response to the application of conditioned media from myoblasts, whereas the mechanical function was reduced.

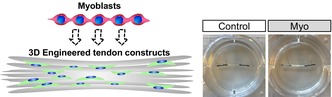

## 1 INTRODUCTION

Tendon injuries and disorders associated with repetitive overuse (tendinopathy) are common musculoskeletal problems, leading to performance difficulties in workers who are exposed to excessive high force and/or repetitive loads and sports athletes (Millar et al., 2021). Tendinopathy can induce chronic pain, regional swelling, increased risk of re‐rupture, and decreased quality of life due to the poor healing capacity of tendons. Unfortunately, although tendons play a central role in human movement, knowledge of the slow intrinsic healing process of the tendon after such injuries is limited, and the molecular mechanism during healing is still not fully investigated (Millar et al., 2021) (Snedeker & Foolen, 2017).

Tendon consists of tendon specific stromal fibroblasts within a mainly type I collagen‐rich extracellular matrix (ECM), and its role is to transmit and buffer mechanical loads between muscle and bone (Kjaer, 2004). Recently, a variety of physiological and biological molecular activities of tendons have been revealed, such as the existence of several types of cell populations (Kendal et al., 2020) (De Micheli et al., 2020), circadian regulation of collagen synthesis (Chang et al., 2020), and autophagy regulations (Montagna et al., 2022). These activities are similar to skeletal muscle (Yoshimoto & Oishi, 2023). Further, some studies have suggested interplay between tendon and skeletal muscle on the functional and physiological aspects of both tissue development and maintenance of musculoskeletal homeostasis (Barin et al., 2019; Connizzo & Grodzinsky, 2018; Delgado Caceres et al., 2021; Frolova et al., 2014; Sato et al., 2014; Subramanian et al., 2018; Tsuchiya et al., 2022, 2023; Yeung et al., 2022). For example, mice deficient in tenogenic genes exhibit lower muscle mass, reduced function, and diminished voluntary running capacity (Frolova et al., 2014) (Delgado Caceres et al., 2021), while an injured tendon leads to skeletal muscle fibrosis and stiffening (Sato et al., 2014). In addition, we have recently reported the importance of interplay between tendon fibroblasts and myoblasts during myogenic differentiation (Tsuchiya et al., 2022). Just as there is influence from tendon to muscle, it has been reported in zebrafish that cellular mechanotransduction responses to muscle contraction alter the differentiation and morphogenesis of cells in the tendon via upregulation of transforming growth factor beta (TGF‐*β*) signaling (Subramanian et al., 2018). In addition, injury of the gastrocnemius muscle in rats alters ECM remodeling of the calcaneal tendon and its functional properties (Barin et al., 2019). These findings indicate that muscle influences tendon homeostasis, and establishing this interplay would help tendon regeneration and development.

It is well known that skeletal muscle releases several factors defined as myokines, which are physiologically active substances with beneficial effects and therapeutic potential for several target tissues, e.g., adipose, liver, or brain (Pedersen et al., 2007). However, the interaction between muscle and tendon in terms of tendon regeneration and development remains to be elucidated. Thus, it is important to understand how cells in skeletal muscle may affect cells in tendon for maintaining their homeostasis during regeneration/development processes.

In this study, we determined the potential of myoblasts for promoting tendon healing, as characterized by collagen production (a proxy for tenocyte differentiation), migration potential, morphology, and mechanical function using two‐dimensional (2D) and three‐dimensional (3D) culture models. The conditioned medium from human myoblasts was applied to human tendon fibroblasts, and their tenogenic progression was evaluated by immunofluorescence staining and functional tests.

## 2 METHODS

### 2.1 Ethical approval

Written and informed consent for donation of excess reconstructive tissues (semitendinosus and gracilis tendon‐muscle tissues) was obtained from 9 donors undergoing anterior cruciate ligament reconstruction (age: 31.3 ± 8.5 years mean ± standard deviation [SD]). All experiments for the present human study were approved by the Ethical Committee of Copenhagen (Ref. H‐3‐2010‐070) and performed in accordance with the Helsinki Declaration II.

### 2.2 Cell isolation and culture

**FIGURE 1:**
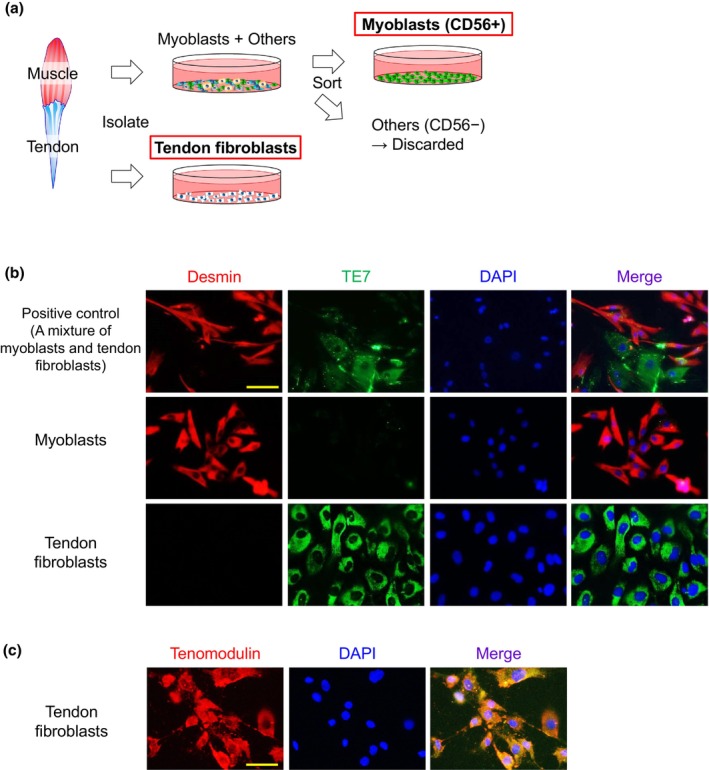
Purification of human myoblasts and tendon fibroblasts. (a) Purification procedure of human muscle and tendon tissue‐derived myoblasts and tendon fibroblasts, respectively, from matching donors. Myoblasts were sorted using anti‐CD56 magnetic beads. Purified myoblasts (CD56+ cells) and tendon fibroblasts were used in this study. (b) Immunostaining of myoblasts and tendon fibroblasts with desmin (red), TE7 (green), and DAPI (blue). Scale bar, 50 μm. (c) Immunostaining of tendon fibroblasts with scleraxis (green), tenomodulin (red), and DAPI (blue). Scale bar, 50 μm.

Myoblasts and tendon fibroblasts were isolated from the same muscle‐tendon tissue unit of each donor (Figure 1(a)) as previously described (Tsuchiya et al., 2023). In brief, muscle tissues were cut into small pieces (<0.1 cm^3^) after removing all visible non‐muscle tissue and digested for 1 h with Skeletal Muscle Basal Medium (PromoCell, Heidelberg, Germany; C‐23260) containing 0.2% type B collagenase (Roche Sigma Aldrich, Darmstadt, Germany; 11,088,815,001) and 0.2% dispase 170 II (Roche Sigma Aldrich; D4693‐1G) at 37°C, in 5% CO_2_. After filtrating through a 100 μm cell strainer (BD Falcon, NJ, USA; 352,360), cells were washed with Human Skeletal Muscle Growth Medium (mGM: myoblasts Growth Medium) (PromoCell; C‐23060) supplemented with 10% of Skeletal Muscle Growth Medium Supplement Mix (PromoCell; C‐39365), 15% fetal bovine serum (FBS) (BioWest, MA, USA; S181H‐500), and 1% L‐Glutamine penicillin–streptomycin (PS) solution (Roche Sigma Aldrich; G6784), and then myoblasts were cultured in T25 flasks.

Tendon tissues were minced into small pieces (<0.5 cm^3^) after removing all visible non‐tendon tissue. The tendon pieces were digested overnight (for 17–24 h) in Dulbecco's Modified Eagle Medium/Nutrient Mixture F‐12 (DMEM/F12; Thermo Fisher Scientific, Hvidovre, Denmark; 21,041,025) containing 0.1% type II collagenase (Worthington Biochemical, NJ, USA; 43D14160) and 20% FBS at 37°C, in 5% CO_2_. After washing with DMEM/F12 supplemented with 10% FBS and 1% PS solution (tGM; tendon fibroblast Growth Medium), the tendon fibroblasts were cultured into T75 flasks. The mGM and tGM were changed every 2–3 days. The passage number of myoblast and tendon fibroblast used in the present study was ≤5.

### 2.3 Sorting of human myogenic cells

Human primary cultured muscle tissue derived cells were purified to obtain myoblasts using anti‐CD56 magnetic beads (Miltenyi Biotec SAS, Paris, France; 130–050–401) referring to previously published methods (Tsuchiya et al., 2023) (Figure 1(a)). Briefly, cultured myoblasts were detached by incubation with a trypsin EDTA solution (Biological Industries, Beit Haemek, Israel; 03–054–1B) diluted 1:2 in phosphate‐buffered saline (PBS) and incubated for 1.5 min, then mGM was added immediately after cell detachment for protection against trypsinization damage of cell surface epitopes. After centrifugation for 6 min at 600*g*, the cell pellet mixed with 170 μL of MACS buffer (Milteneyi Biotec; 130–091–221) and 35 μL of the anti‐CD56 magnetic beads was incubated at 5°C for 15 min. Then, the cell suspension was diluted with 5 mL MACS buffer, and centrifuged for 6 min at 600*g*. The pellet was resuspended in 1 mL MACS buffer, and the solution was passed through a pre‐separation filter (Miltenyi Biotec; 130–041–407) and a large cell column (Miltenyi Biotec; 130–042–202) held in a MiniMACS separator (Miltenyi Biotec; 130–090‐312). The cells trapped by CD56+ magnetic beads in the column were collected into a CD56+ fraction tube after separating from the magnet. The CD56+ cells were recovered in mGM for the experiments. Cell populations were assessed by immunostaining for desmin and TE7 (Figure 1(b)).

### 2.4 Forming 3D tissue‐engineered human tendon constructs

Tendon constructs were assembled from the primary human tendon fibroblasts as described previously with minor modifications (Bayer et al., 2010). A 6‐well plate was coated with silicone (mixture of 184 Silicone Elastomer Base and Silicone Curing Agent, Dow‐Chemicals, Michigan, USA; DOW SYLGARD 184) and incubated to set at 55°C for 48 h. In the middle of each well, two short silk sutures (Bunzl Healthcare, London, UK; W328H) were pinned onto the plates using stainless steel minuitiens insect pins (0.1 mm diameter) (Fine Science Tools, California, USA; 26,002–10) with a distance of 10 mm between sutures. After the plates were sterilized by immersion in 100% ethanol for 60 min, all wells of the plates were washed with 5 mL of sterile PBS.

Tendon fibroblasts were suspended in a mixture containing 200 μL of human plasma fibrinogen (Roche Sigma Aldrich; F3879‐1G) at 20 mg/mL, 10 μL of aprotinin (Roche Sigma Aldrich; A6103‐100MG) at 10 μg/mL, 4 μL of human plasma thrombin (Roche Sigma Aldrich; T4393‐100UN) at 3 U/mL, and 600 μL of tendon construct medium (tCM) (DMEM/F12 containing 10% FBS, 20 mM L‐ascorbic acid 2‐phosphate (Roche Sigma Aldrich; A8960‐5G), 10 mM L‐proline (Roche Sigma Aldrich; P5607‐25G)) to a final cell density of 2 × 10^5^ cells per well. The 814 μL mixture was seeded quickly into each well and the 6‐well plate was incubated in the cell incubator at 37°C, in 5% CO_2_ for 30 min. Then, 3 mL of tCM was added. The tCM was changed every other day. All tendon constructs were cultured for 21 days.

### 2.5 Collection of conditioned media

**FIGURE 2:**
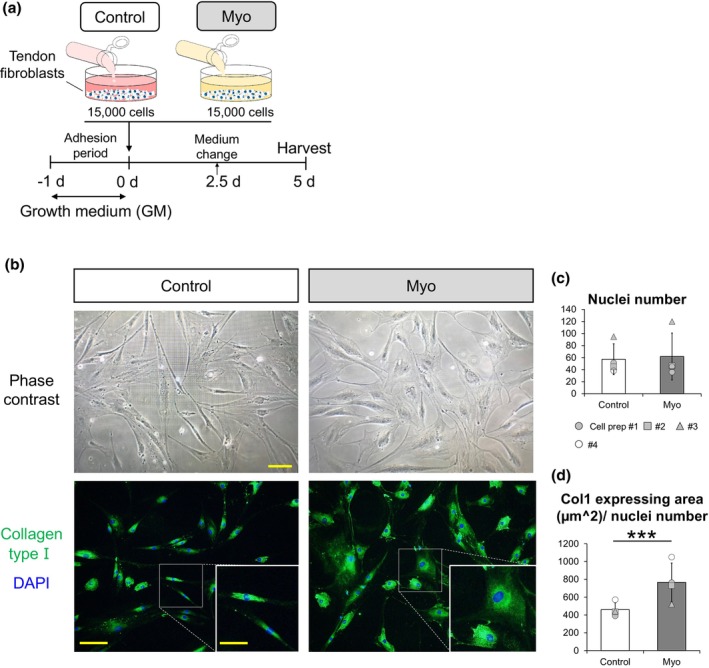
Nuclei number and collagen type I immunostaining in tendon fibroblasts. (a) Schematic illustration of the experimental procedure. Tendon fibroblasts were cultured in Control and Myoblasts‐derived (Myo) conditioned media for 5 days. (b) Representative phase contrast and immunostaining images of tendon fibroblasts with collagen type I (green) and DAPI (blue). Scale bar for phase contrast, 50 μm. Scale bar for immunostaining, 100 μm; Scale bars of the boxed area with higher magnification, 200 μm. (c) Quantification of number of nuclei per image. (d) Quantification of collagen I positive area normalized to number of nuclei per image. *N* = 4 in each condition. Matching symbols correspond to tendon fibroblasts from the same donor. ***p < 0.005 vs. Control. Values are means ± SD.

**FIGURE 3:**
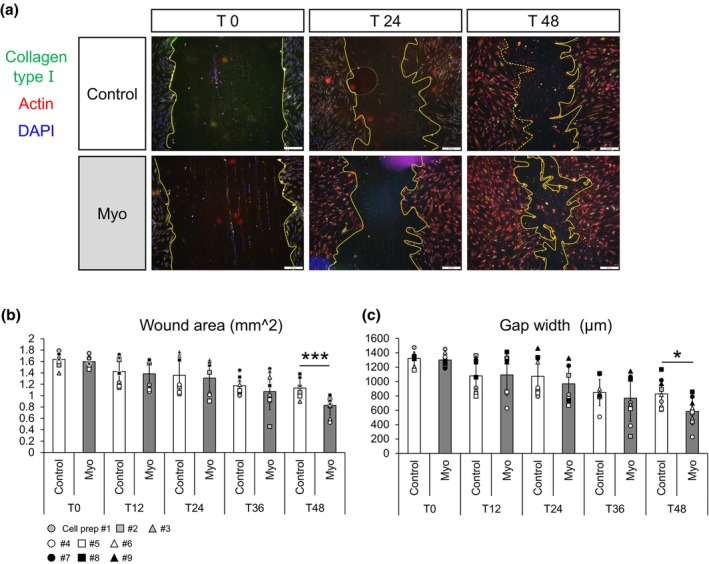
Migration potential of tendon fibroblasts. (a) Representative immunostaining images of tendon fibroblasts with collagen type I, actin, and nuclei (DAPI). Scale bar for immunostaining, 200 μm. The yellow dashed line in the pictures represents the edges of cells. (b) Wound area over time. (c) Gap width over time. *N* = 9 in each condition. Matching symbols correspond to tendon fibroblasts from the same Donor. *p < 0.05 vs. Control. ***p < 0.005 vs. Control. Values are means ± SD.

**FIGURE 4:**
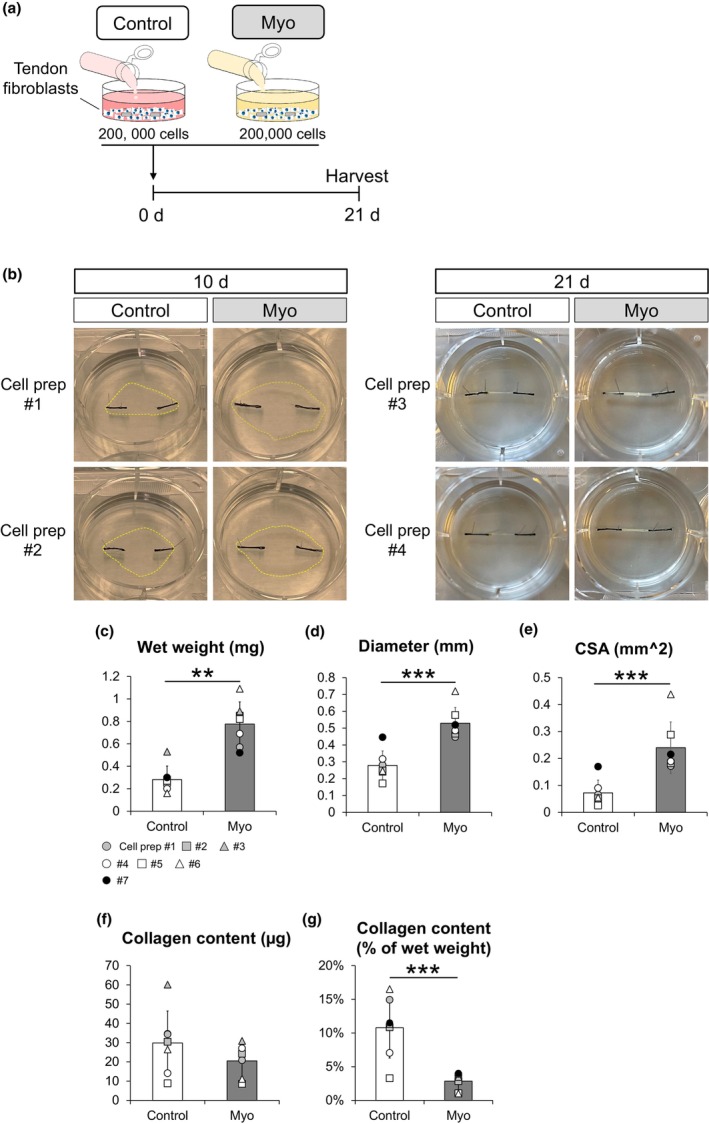
Morphology and collagen content of 3D tissue‐engineered human tendon constructs. (a) Schematic illustration of the experimental procedure. Tendon fibroblasts were cultured in Control and myoblasts‐derived (Myo) conditioned media for 21 days. (b) Representative bright field images of tendon constructs at 10 and 21 days. The yellow dashed lines in the pictures at 10 days indicate fibrin gel contracted by tendon fibroblasts. (c) Wet weight of tendon constructs. (d) Diameter of tendon constructs. (e) CSA of tendon constructs. (f) Collagen content of tendon constructs. (g) Collagen density as % of wet weight in tendon constructs. *N* = 7 in each condition. Matching symbols correspond to tendon constructs from the same donor. **p < 0.01 vs. Control. ***p < 0.005 vs. Control. Values are means ± SD.

**FIGURE 5:**
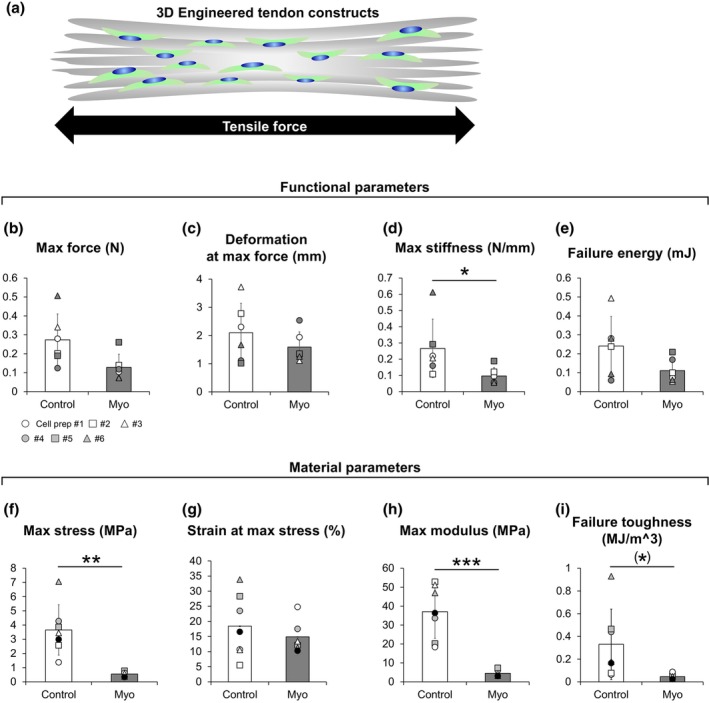
Functional and material properties of tissue‐engineered tendon constructs. (a) Schematic illustration of 3D engineered tendon constructs cultured for 21 days. (b) Max force. (c) Deformation at max force. (d) Max stiffness. (e) Failure energy. (f) Max stress. (g) Strain at max stress. (h) Max modulus. (i) Failure toughness. *N* = 7 in each condition. Matching symbols correspond to tendon constructs from the same donor. *p < 0.05 vs. Control. **p < 0.01 vs. Control. ***p < 0.005 vs. Control. Trends (0.05 ≤ *p* < 0.1) indicated in brackets. Values are means ± SD.

Conditioned media containing myoblast derived factors were obtained as previously described (Yeung et al., 2020). Myoblasts (3 × 10^5^ per well, 6‐well plate) were cultured until 80% confluency in mGM, and then the mGM was replaced with 1 mL of DMEM/F12 containing 1% PS as a serum free media (SFM). After incubation for 24 h, the media were collected and centrifuged at 600*g* for 6 min after which the supernatants were stored at ₋80°C until the experiment. Control supernatants were collected from SFM added to empty wells (without cells) of a 6‐well plate incubated in the same way as the myoblast cultures. Before applying to tendon fibroblasts, the supernatants were diluted 1:1 with tGM for the 2D cell culture experiments (Figures 2 and 3). The supernatants were diluted 1:1 with tCM for the 3D cell culture experiments (Figures 4 and 5). All diluted supernatants were used as conditioned media in the present study.

### 2.6 Cell proliferation and collagen area

Tendon fibroblasts were seeded onto a 24‐well plate containing a sterilized 12 mm glass coverslip with a cell growth area of 1.13 cm^2^ (VWR, Darmstadt, Germany; 631–1577) at a density of 1.5 × 10^4^ cells per well. All cells were initially incubated in tGM for 12 h for sufficient adhesion before applying the conditioned media. The tendon fibroblasts were washed with tGM once and then incubated in the respective conditioned media for 5 days. All conditioned media were changed at 2.5 days (Figure 2(a)). Immunofluorescence images stained for collagen type I and nuclei (4,6‐diamidino‐2‐phenylindole, DAPI) were captured at 5 days. Five images (center, top, left, bottom, and right sides) with 10× magnification on a BX51 Olympus microscope (Olympus Deutschland, Hamburg, Germany) were counted per coverslip using Fiji software (NIH, Bethesda, MD, USA; version 1.52). Cell proliferation assay was evaluated by counting of DAPI+ nuclei number. Collagen production area assay was evaluated by calculating the ratio of collagen type I expressing area per DAPI+ nuclei number.

### 2.7 Migration assay

Tendon fibroblasts were seeded onto a 12‐well plate containing a sterilized 18 mm glass coverslip with a cell growth area of 2.54 cm^2^ (VWR, Darmstadt; 631–1580) at a density of 1.5 × 10^4^ cells per well. All cells were initially incubated in tGM for 24 h for sufficient adhesion before applying the conditioned media. A P1000 tip was used to create a scratch uniaxially in each well. After the scratching, all wells were washed with PBS to remove detached cells. The tendon fibroblasts were incubated in the respective conditioned media for 48 h. Coverslips were fixed at 0, 12, 24, 36 and 48 h after scratching (referred to as T0 to T48) and analyzed by immunofluorescence. Cells were stained for collagen type I, actin, and nuclei as described below. For the quantification of cell migration, the wound area and wound gap width were measured. Three images (top, center, and bottom) with 4× magnification on a BX51 Olympus microscope (Olympus) were collected per coverslip. The wound area (cell free area) was traced automatically using Fiji software. For each image, the gap width was a mean of measurements at two locations. To determine the two locations, diagonal lines were automatically drawn onto the image through the center of the gap and the four points at which the two diagonal lines crossed with the two wound edges were where the width was measured.

### 2.8 Immunofluorescences staining

All cells for immunofluorescence staining were fixed using Histofix (Histolab, Gothenburg, Sweden; 01000) for 8 min and permeabilized for 15 min with 0.01% Triton in 0.05 M Tris‐buffered saline (TBS). All cells were incubated with appropriate primary antibodies (see below) in blocking buffer containing 1% bovine serum albumin (Roche Sigma Aldrich; A3912‐100G) in 0.05 M TBS at 4°C overnight and visualized using goat anti‐mouse Alexa Fluor 488 and goat anti‐rabbit Alexa Fluor 568‐conjugated secondary antibodies (Thermo Fisher Scientific; A11029 and A11036, respectively). For nuclear staining, DAPI‐containing mounting medium (Thermo Fisher scientific; P36931) was used. All images were captured using a camera (DP71; Olympus) on a BX51 Olympus microscope, and digital images were taken using the software (cellSens 1.14; Olympus). For collagen staining assay (Figure 2), five non‐overlapping images (center, top, left, bottom, and right sides of the cover glass) were captured at 10x magnification. For migration assay (Figure 3), three non‐overlapping images along the scratched line were captured at 4× magnification using the same microscope. All acquired digital images were quantified using Fiji software. The following antibodies were used: rabbit anti‐desmin (Abcam, Cambridge, UK; AB32362); mouse anti‐TE7 (Merck, Darmstadt, Germany; CBL271) goat anti‐tenomodulin (Santa Cruz biotechnology, Dallas, USA; SC49325); mouse anti‐collagen type I (Roche Sigma Aldrich; C2456); rabbit anti‐actin (Roche Sigma Aldrich; A2066).

### 2.9 Mechanical testing

Tensile testing of the engineered tendon constructs was performed at 21 days after cell seeding (Figure 4(a)). The test was conducted using a PC‐driven micromechanical rig with a liquid chamber (20 N load‐cell; Deben, Suffolk, UK). A stereoscopic microscope (SMZ1000, Nikon, Tokyo, Japan) equipped with a digital camera (SC50, Olympus), which was used for recording during the test to verify the length and monitor the rupture site of the constructs. The tendon construct ends attached to silk suture were placed onto each specimen plate of the mechanical rig and glued with cyanoacrylate. After curing the glue for 15–30 min, mechanical testing was performed. During all preparation, the samples were wrapped in culture medium‐soaked gauze to avoid drying. The samples were stretched to the onset of force at which point images of the construct diameter and length were captured using the microscope. The construct diameter was measured in four different places and a cross‐sectional area (CSA) was calculated based on assumption of a circular cross‐section. After that, the constructs were stretched at 4 mm/min of speed until failure. For each test, functional parameters: max force, deformation at max force, max stiffness (slope at the steepest point of the curve), and failure energy (area under the force‐deformation curve up to the max point), as well as the equivalent material parameters: max stress, strain at max stress, max modulus, and failure toughness were determined.

### 2.10 Hydroxyproline assay

Collagen content in 3D culture model was estimated from hydroxyproline quantification as previous described (Heinemeier et al., 2016). In brief, tendon constructs were hydrolyzed in 6 M HCl at 110°C overnight, dried and reconstituted in 310 μL acetate‐citrate buffer. Hydroxyproline content in samples and standards was then determined by colorimetric reaction with 4‐dimethylaminobenzaldehyde, and expressed as μg of collagen assuming a 13.5% hydroxyproline content by weight. Collagen density was also determined as a % of wet weight.

### 2.11 Statistical analysis

Data are shown as mean ± SD. A Wilcoxon matched‐pairs signed‐rank test was applied to identify significant differences between Control (unconditioned media) and Myo conditions (myoblast conditioned media) for immunofluorescence staining and mechanical data. Significance and trend were set at *p* < 0.05 and *p* < 0.1, respectively. All data were analyzed using SPSS version 28.0.1.1 (IBM Corp., Armonk, NY, USA).

## 3 RESULTS

### 3.1 Purification of human myoblasts and tendon fibroblasts

The purification of human myoblasts and tendon fibroblasts was confirmed by immunostaining (Figure 1). Myoblasts sorted by anti‐CD56 magnetic beads and isolated tendon fibroblasts were confirmed by immunostaining for desmin, a myoblast marker, and TE7, a fibroblast marker (Figure 1(b)). Further, tendon fibroblast purity was confirmed by immunostaining for tenomodulin as tendon cell marker (Figure 1(c)).

### 3.2 Conditioned media derived from human myoblasts extend collagen expressing area of human tendon fibroblasts

To examine how conditioned media derived from human myoblasts influence proliferation of human tendon fibroblasts and their production of collagen type I, which is the main component of tendon, we compared the areas of collagen type I staining in Myo with Control conditions (Figure 2(a)). There was no significant difference in the number of DAPI+ nuclei per image between conditions (Figure 2(b), (c)). However, the area of collagen type I positive staining normalized to the number of DAPI+ nuclei per image was significantly larger in the Myo condition than in the Control condition (Figure 2(b), (d)).

### 3.3 Conditioned media derived from human myoblasts facilitate migration of human tendon fibroblasts

A larger collagen type I expressing area in the Myo condition (Figure 2) could contribute to making a scaffold supporting migration of the tendon fibroblasts. We therefore tested the migration properties of tendon fibroblasts treated with conditioned media from myoblasts. Firstly, we confirmed that the wound areas in both conditions were not significantly different immediately after scratching at T0 (Figure 3). The wound area did not differ between the two conditions at T12, T24, or T36. However, the wound area significantly decreased in the Myo condition compared to the Control condition at T48 (Figure 3(a),(b)). In line with this, the gap width did not show significant differences between conditions at T12, T24, or T36, but it was significantly shorter in the Myo condition than in the Control condition at T48 (Figure 3(a),(c)).

### 3.4 Conditioned media derived from human myoblasts suppress the collagen synthesis and mechanical properties of 3D tissue‐engineered human tendon constructs

Wet weight, diameter, and CSA values of the tendon constructs were significantly greater in the Myo condition than in the Control condition (Figure 4(b)–(e)). However, collagen content in the tendon constructs did not show a significant difference between conditions (Figure 4(f)) and the collagen density was significantly lower in the Myo condition than in the Control condition (Figure 4(g)).

As a functional test, we determined the mechanical properties of the tendon constructs (Figure 5(a)). Contrary to the morphology of the tendon constructs in the Myo condition, max force, deformation at max force, and failure energy did not differ significantly between conditions (Figure 5(b), (c), and (e)), and max stiffness was even significantly lower in the Myo condition compared to the Control condition (Figure 5(d)). Combined with the increased CSA, this resulted in lower tissue mechanical quality in the Myo condition as seen from the material properties where max stress and max modulus were significantly lower (Figure 5(f) and (h)). Further, failure toughness tended to be lower in the Myo condition than in the Control condition (Figure 5(i)). These results suggest that although tendon constructs in the Myo condition were thicker, ECM formation and consequently the functional properties did not follow at 21 days after seeding.

## 4 DISCUSSION

This is the first study to describe an impact of myoblasts on tenogenic progressions in cells derived from matched human muscle and tendon tissues. The main finding of this study was that conditioned medium from human myoblasts led to extended collagen area and greater migration potential of the tendon fibroblasts, as well as hypertrophy of the tissue engineered human tendon constructs. However, the tendon constructs exhibited lower collagen density and weakened mechanical properties compared to the Control condition.

Our results indicated that myokines, which are defined as secretion factors from skeletal muscle (Pedersen et al., 2007), released by the myoblasts induced several tenogenic responses. It has been reported that cells in the extrinsic compartment of the tendon, which comprise the paratenon, epitenon, and endotenon, recruit extensive neo‐vascularization, enabling delivery of several growth factors for tendon healing (Snedeker & Foolen, 2017). Since skeletal muscle is located adjacent to tendon, myokines taken into tendon could support tendon healing or development in humans.

In 2D culture, a larger area of collagen type I staining in the Myo condition was found in the present study, indicating that myoblast derived factors influence the early formation and assembly of collagen type I in tendon fibroblasts. Although we did not determine the specific factors, several candidates exist; IL‐6 is reported to stimulate tendon collagen synthesis and has been detected by a cytokine array in conditioned media derived from C2C12 myoblasts (Andersen et al., 2011) (Ghebes et al., 2018). In addition, a microRNA (miR) 206 in extracellular vesicles released from satellite cells (progenitor of myoblast) has been shown to regulate collagen synthesis in muscle fibrogenic cells during muscle regeneration to prevent excessive ECM deposition (Fry et al., 2017). Thus, myoblast derived inflammatory cytokines and miRs could appropriately control collagen production in the present study. As a next step, it would be important to fully investigate the secretory profile of myoblasts by proteomics in the future.

We also observed an improved migration potential of the tendon fibroblasts in Myo condition, suggesting that myoblast‐derived factors are also responsible for enhancing cell migration. It has been reported that C2C12 myoblasts released chemokine ligands CCL1, CCL5, and vascular endothelial growth factor as chemotactic cytokines involved in cell migration (Ghebes et al., 2018). We presume that promoted tendon fibroblast migration potential in the Myo condition reflects not only larger collagen area but potentially also activities of chemotactic cytokines.

In the 3D culture model, we observed larger tendon constructs in the Myo compared to the Control conditions, but this did not correspond to any larger collagen content, resulting in a reduction of both functional and material properties. In general, formation of tissue engineered tendon constructs requires both optimal collagen synthesis and development of matrix supporting proteins to stabilize between collagen fibrils (Avery & Bailey, 2005) and to induce high mechanical strength (Herchenhan et al., 2013). The present findings suggest that impaired accumulation of collagen in the construct ECM was a significant contributor to the reduced mechanical function. However, the reduction in material properties was even greater than the reduction in collagen density and the functional properties were impaired even though the total collagen content was not significantly different between conditions. This suggests that additional factors may also contribute to the reduced mechanical quality such as; (i) changes in matrix supporting proteins, (ii) shifting proteoglycan contents, (iii) reduced fibrillar cross‐link formation, or (iv) disorganization of synthesized collagen (Snedeker & Foolen, 2017) (Chen et al., 2012). Firstly, to our knowledge, there are no studies on the effect of myoblast derived factors on matrix supporting protein productions, such as decorin, fibromodulin, and tenascin‐X, which are essential for collagen fibril formation (Fleischmajer et al., 1991; Kalamajski & Oldberg, 2007; Orgel et al., 2009; Svensson et al., 1999). Thus, our data indicate a possibility that a number of these matrix supporting proteins may be impaired. Secondly, the increased wet weight, diameter, and CSA of tendon constructs in the Myo condition could be related to a shifting of proteoglycan content from small leucine‐rich proteoglycans to larger hydrophilic proteoglycans, which is associated with increased water content and tendon thickening during tendon healing (Docking et al., 2015). A shift in proteoglycans may also directly affect the mechanical properties since small leucine‐rich proteoglycans contribute to the viscoelastic properties of the mature tendon (Millar et al., 2021; Thorpe et al., 2013). Therefore, factors derived from myoblast may induce changes to the proteoglycan profile, leading to increased water content in the tendon constructs and poor force transmission. Another contributing factor may be a lack of lysyl oxidase (LOX), which is responsible for fibrillar cross‐link formation in tendons (Herchenhan et al., 2015) (Magnusson & Kjaer, 2019). Chronic injection with IL‐6 protein in rat tendons has been shown to suppress expression of LOX (Katsma et al., 2017). Since IL‐6 is produced from C2C12 myoblasts (Ghebes et al., 2018), it is possible that myoblasts‐derived IL‐6 stimulates collagen production while suppressing the production of LOX, resulting in reduced quality and potentially poor retention of the collagen in the ECM of tendon construct. Lastly, reduced organization of the synthesized collagen may also contribute to a reduction of the mechanical function in tension. Tendinopathic tendon exhibits reduced collagen fibril organization, such as distortions in collagen fibril bundle alignments, an increase in small diameter collagen fibrils, or a separation between cells and ECM (Pingel et al., 2014). These conditions are considered to be controlled by dysregulation of factors associated with signaling pathways (ERK, Wnt, and TGF‐*β*) and transcriptional factors (SCX and EGR1) of tendon fibroblasts (Millar et al., 2021) (Yoshimoto & Oishi, 2023) (Guerquin et al., 2013), and it is possible that myoblasts derived factors affect these pathways as well. Taken together, we hypothesize that the role of the present myoblast derived factors is only to induce hypertrophy (greater wet weight, diameter, and CSA) of tendon while other growth factors are required to ensure appropriate matrix formation and retention as well as supporting protein production, proteoglycan composition, cross‐linking, or organization of collagen fibrils.

The present study has some important limitations. Firstly, we could not technically develop a co‐culture model, which allowed constant crosstalk between myoblasts and tendon fibroblasts during formation of tendon constructs. Additionally, the conditioned media was derived from myoblasts but not from differentiated myotubes. Our group has previously found distinct factors between myoblasts and tendon fibroblasts using proteome analysis (Yeung et al., 2020), but in the future, the effect of factors derived from mature myotubes on tenogenic progression should be examined. Secondly, the conditioned media may not have been completely matched due to nutrient consumption by myoblasts in the Myo condition compared to the Control condition. Nevertheless, supernatants obtained from both conditions were diluted with fresh tGM or tCM to ensure nutrient availability. It is unclear how the basic nutrient difference may contribute to tenogenic progressions, but in light of the added fresh medium, we believe that myoblast derived factors were the primary driver of the present findings. In support of this, there is evidence that media from C2C12 myoblasts contain some cytokines and growth factors that induces tenogenic gene expression (scleraxis and tenomodulin) when compared to media from serum‐free conditions in 2D culture (Ghebes et al., 2018), which would support the role for muscle‐derived factor in regulating tendon differentiation.

In conclusion, we have revealed that conditioned media derived from myoblasts extended collagen area and enhanced cell migration in a 2D culture model, while contributing to a lower collagen density and a loss of mechanical function in a 3D tissue engineered human tendon construct model.

The findings of the present study provide fundamental information on the interplay between muscle and tendon during tendon healing and development.

## AUTHOR CONTRIBUTIONS

Y.T designed and performed the experiments, interpreted and analyzed the data, and wrote the manuscript. C.‐Y.C.Y designed experiments, provided technical support, interpreted the data and reviewed the manuscript. R.B.S designed the experiments, provided technical support, interpreted the data, and reviewed the manuscript. M.K acquired funding, interpreted data, and reviewed the manuscript.

## FUNDING INFORMATION

This work was supported by the Uehara Memorial Foundation for Overseas postdoctoral fellowships. This work was also supported by the Danish Medical Research Council (FSS) (0134‐00028B), Lundbeck Foundation (R198‐2015‐207) and Novo‐Nordisk Foundation (NNF16‐0C0022846).

## CONFLICT OF INTEREST STATEMENT

The authors declare no conflict of interest.

## Data Availability

The data that support the findings of this study are available from the corresponding author upon reasonable request.
